# A DFT study of the gallium ion-binding capacity of mature *Pseudomonas aeruginosa* biofilm extracellular polysaccharide

**DOI:** 10.1371/journal.pone.0287191

**Published:** 2023-06-14

**Authors:** Oliver J. Hills, Zuzanna Poskrobko, Andrew J. Scott, James Smith, Helen F. Chappell

**Affiliations:** 1 School of Food Science & Nutrition, University of Leeds, Woodhouse Lane, Leeds, United Kingdom; 2 School of Chemical & Process Engineering, University of Leeds, Woodhouse Lane, Leeds, United Kingdom; University of Delhi, INDIA

## Abstract

Intravenous gallium therapy is a non-antibiotic approach to limit *Pseudomonas aeruginosa* biofilm proliferation, by outcompeting iron for siderophore binding. Gallium therapy represents a viable therapeutic strategy for cystic fibrosis (CF) patients harbouring mucoid *P*. *aeruginosa* biofilm lung infections. Siderophore deficient *P*. *aeruginosa* isolates still demonstrate a hindered biofilm proliferation when exposed to gallium but it is currently unknown whether exogenous gallium has any disruptive influence on the exopolysaccharide (EPS), the major mucoid *P*. *aeruginosa* CF lung biofilm matrix component. To that end, Density-Functional Theory (DFT) was deployed to assess whether gallium (Ga^3+^) could be substituted into the mature mucoid EPS scaffold in preference of calcium (Ca^2+^)—the native EPS cross-linking ion. Removal of the stable, bound native calcium ions offers a large enthalpic barrier to the substitution and the mature EPS fails to accommodate exogenous gallium. This suggests that gallium, perhaps, is utilising a novel, possibly unknown, ferric uptake system to gain entry to siderophore deficient cells.

## Introduction

*Pseudomonas aeruginosa* is the most prevalent and problematic pulmonary pathogen in cystic fibrosis (CF) patients [1, 2], easily establishing chronic infections in the thick, viscous mucus present within the CF lung [3], and currently accounting for the majority of the mortality seen in CF disease [1].

The chronicity of *P*. *aeruginosa* infections in the CF lung is attributed to this bacterium’s ability to form a mucoid biofilm [4]. Such biofilms possess a matrixome composed primarily of the exopolysaccharide (EPS) alginate [5, 6], encompassing both mannuronate and guluronate, with exclusive mannuronate acetylation at C2 or C3 positions [7–9]. Within the pH range of the CF lung (6.85–7.65) the mannuronic acid and guluronic acid residues of the alginate chains will have deprotonated carboxylic acid groups, providing environments that are potentially suitable for cationic binding [10, 11]. We therefore find that the EPS alginate chains are electrostatically cross-linked by Ca^2+^ ions, which are significantly elevated in concentration in the CF lung [12], to form highly thermodynamically stable aggregates [13]. Mature mucoid *P*. *aeruginosa* CF biofilms retard antimicrobial penetration, affording the biofilm-enveloped bacteria resistance to nearly all clinically relevant classes of antibiotics [14]. In light of this, recent impetus has been directed towards devising novel non-antibiotic treatments that are able to eradicate chronic CF mucoid *P*. *aeruginosa* infections. A recent systematic review conducted by Firoz *et al*., evaluating 30 research studies reporting data on iron chelating therapies, concluded that novel therapeutic approaches targeting iron uptake have potent anti-biofilm properties and represent a viable solution for treating mucoid CF *P*. *aeruginosa* biofilms [15]. Iron (Fe^3+^) is critical for mediating *P*. *aeruginosa* growth, pathogenicity, biofilm formation and virulence in the CF lung [16], and iron uptake is facilitated through siderophores with high ferric binding affinities (binding constants up to 10^50^ M^-1^) [17]. These molecules are capable of sequestering free iron from the immediate bacterial environment and are even capable of stripping iron from host proteins [17]. *P*. *aeruginosa* cells cannot obtain sufficient levels of iron to facilitate biofilm proliferation and maintenance by passive diffusion alone and siderophores therefore play a critical role in the establishment of mature, structurally heterogeneous, biofilm matrices [16].

Within the CF lung, iron levels are elevated by 700 *μ*g/L relative to non-CF controls [12] and, given the observation that sputum microalbumin is also elevated in CF patients, significant vascular leakage as part of the inflammatory process in CF disease can be inferred [18]. *P*. *aeruginosa* utilises pyochelin as its primary siderophore in mediums where iron levels are not limited [16] and transcriptional analysis of pyochelin genes shows high expression during the early stage of bacterial growth in CF sputum [19]. This indicates that pyochelin is deployed to sequester iron upon colonisation of the CF lung *in vivo*. Pyochelin possess phenolate, amine and carboxylate binding sites that collectively bind Fe^3+^. Electrospray ionisation mass spectrometry has shown the existence of both the monochelate and bischelate ferric complexes ([FeL]^+^ and [FeL_2_]^-^ respectively, where L = pyochelin) at physiological pH [20]. In extracellular medium, however, it is the 1:1 monochelate complex that dominates, binding Fe^3+^ in a 4-coordinate chelate complex, with the two vacant coordination sites occupied by hydroxide and/or water [21], although these additional ligands are not required for cellular recognition and uptake [22].

Outcompeting siderophore systems for iron sequestration appears, therefore, to be a viable strategy to treat mucoid *P*. *aeruginosa* CF biofilms *in vivo*. For example, it has been shown that iron sequestration, using exogenous lactoferrin, induces a *P*. *aeruginosa* twitching mobility, promoting surface locomotion and making the bacterium unable to settle onto the substratum and form a biofilm [23]. In addition, chelation of iron using conalbumin is able to drastically reduce *P*. *aeruginosa* biofilm proliferation on cells expressing the ΔF508-CFTR mutation *in vitro*, indicating that high iron levels play a role in biofilm proliferation [24]. Furthermore, in iron-deplete environments, rhamnolipid biosynthesis is upregulated 7–10 fold in the initial stages of *P*. *aeruginosa* biofilm development, contributing to the formation of thin, homogenous, biofilm matrices which are easy to clear [25].

Recently, intravenous gallium nitrate Ga(NO_3_)_3_ therapy has emerged as a novel therapeutic strategy that is both safe and efficacious, inhibiting *P*. *aeruginosa* growth in the lungs of CF patients [26]. It has been shown that only micromolar concentrations of gallium are required to completely inhibit *P*. *aeruginosa* growth on CF sputum [26, 27] and on human serum extracted from CF patients [28]. In addition to slowing bacterial growth, gallium is effective against *P*. *aeruginosa* cells embedded deep in the centre of the biofilm matrix [27] and is even able to completely block *P*. *aeruginosa* biofilm proliferation [27, 29]. Encouragingly, following gallium nitrate therapy, the regain in lung function, in CF patients harbouring mucoid *P*. *aeruginosa* lung infections, is comparable to that observed when using vigorous conventional antibiotic therapy, but without the development of resistance [26]. Furthermore, studies exploring siderophore activity in relation to gallium exposure have found a positive correlation with anti-growth activity, suggesting an interesting association between gallium activity and siderophore quenching [30].

Gallium nitrate is a source of Ga^3+^ ions and octahedral Ga^3+^ possesses an ionic radius of 0.620 Å, which is very similar to the ionic radius of (high-spin) octahedral Fe^3+^ (0.645 Å) [31]. This geometrical similarity permits Ga^3+^ to mimic the behaviour of Fe^3+^ in the so-called “Trojan Horse” mechanism. Ga^3+^ is mistaken for Fe^3+^ and sequestered by siderophores, subsequently interfering with physiological processes dependent upon Fe^3+^ [32]. In addition to siderophore quenching, gallium exposure also down-regulates *pvds* expression, a gene controlling the biosynthesis of siderophores under iron-limited conditions [26, 27].

Importantly however, *P*. *aeruginosa* mutants deficient in pyochelin and pyoverdine continue to display reduced biofilm proliferation [27], suggesting that the therapeutic activity of gallium does not solely rest on the Trojan Horse competition for siderophore binding. In fact, in mucoid *P*. *aeruginosa* CF clinical isolates, inactivation of the pyochelin and pyoverdine siderophore uptake systems did not have any effect on the resistance profile of exogenous gallium nitrate therapy [33]. Furthermore, gallium-siderophore complexes are translocated across the *P*. *aeruginosa* bacterial cell envelope at far slower rates (35 times slower) compared to iron-siderophore complexes [34], which suggests that, potentially, gallium can exploit another entry mechanism which is more efficient, or can reduce biofilm matrix proliferation directly through structural alterations to the EPS at the atomistic-scale.

This raises the question of whether gallium is having a direct effect on the EPS architecture, and it is this that is weakening the biofilm structure. This is a phenomenon yet to be tested either *in vitro* or *in vivo*. Yet, encouragingly, Ga^3+^ ions have been observed to bind favourably to alginate scaffolds in theoretical studies based on Density-Functional Theory (DFT) [35] as well as in energy dispersive spectroscopy (EDS) mapping of gallium-alginate hydrogels [36].

To that end, the aim of this work, was to investigate whether Ga^3+^ ions could be accommodated by the mature mucoid *P*. *aeruginosa* EPS scaffold, displacing calcium cross-links and weakening the overall biofilm structure. Previously within the group, atomistic models of the mucoid *P*. *aeruginosa* EPS were developed encompassing the key *in vivo* structural motifs unique to mucoid *Pseudomonas*. These models critically implicated calcium ions (Ca^2+^) in highly thermodynamically stable, cation-induced EPS aggregation and, thus, chronic infection [13]. Calcium ions adopt [13] permanent junction points (cross-linking sites) between EPS scaffolds, giving rise to mechanically stable network structures possessing high storage and Young’s moduli [37, 38]. Recently, it has been demonstrated that mucoid *P*. *aeruginosa* biofilm proliferation is, in fact, calcium-dependent with the calcium cross-linked EPS matrix supporting a diffuse cell arrangement and calcium-EPS interactions being solely responsible for EPS scaffold morphology [39]. Indeed, recent molecular dynamics simulations performed within the group show that the diversity in the Ca^2+^ chelation geometry dictates EPS macrostructure [40].

Mucoid *P*. *aeruginosa* biofilm matrices stiffen over time, transitioning from viscoelastic liquids at their inception to a viscoelastic gel once mature and these matrices are resistant to further changes and remodelling over long time-scales [41]. Conversely, gallium-alginate scaffolds are hard, brittle and prone to degradation through gallium-for-sodium cation exchange, displaying fast degradation kinetics *in vitro* [29, 36]. Therefore, if gallium ions could be accommodated by the EPS, in preference of the native calcium ions, then potentially this would create a scaffold morphology more susceptible to physiological softening through sodium-ion exchange, thus reducing matrix proliferation. Here we employ first principles Density Functional Theory optimization to assess the thermodynamic feasibility of this EPS gallium for calcium exchange.

## Materials & methods

### Computational details

All Density-Functional Theory (DFT) calculations were performed using the plane-wave pseudopotential code, CASTEP [42], using a convergence tested cut-off energy of 900 eV, and a Monkhorst-Pack k-point grid of 1 x 1 x 1 to sample the Brillouin zone [43]. On-the-fly ultrasoft pseudopotentials [44] and the PBE-TS exchange-correlation functional were employed [45, 46]. The SCF tolerance for the electronic minimisations was set to 1×10^−7^ eV Atom^-1^ and the energy, force and displacement tolerances for the geometry optimisations were set to 1×10^−5^ eV Atom^-1^, 0.03 eV Å^-1^ and 1×10^−3^ Å respectively. Following each geometry optimization, Mulliken bond populations were calculated to classify the nature of bonding in each of the complexed structures [47]. All atomistic models were created and visualised using CrystalMaker^®^ [48].

Chemical potentials for sodium, calcium and gallium were calculated by their respective 0 K energy per atom from the pure metals in their lowest energy configurations, namely, hexagonal (HCP) sodium, cubic (BCC) calcium and orthorhombic (alpha) gallium.

### Molecular models

The mucoid *P*. *aeruginosa* EPS encompasses two distinct polyuronate fractions, formed from mannuronate (M) and guluronate (G) residues. These unique fractions are, specifically, acetylated copolymeric β-D-mannuronate-α-L-guluronate (PolyMG) and acetylated poly-β-D-mannuronate (PolyM). Both the PolyMG and PolyM scaffolds bind calcium (Ca^2+^) ions to form thermodynamically stable chelate complexes, facilitating EPS cross-linking and aggregation [13, 37, 38, 49]. Molecular models of 2-chain calcium cross-linked PolyMG and PolyM EPS scaffolds have been developed previously within the group [13] with the most thermodynamically stable calcium cross-linking sites across the scaffolds being identified. The scaffolds were charge saturated, which gave rise to tightly aggregated matrices stabilised through electrostatic interactions [16]. These models are used here as the model systems to study calcium-for-gallium cation exchange into the EPS.

Given that calcium ions are implicated in the establishment of mature mucoid *P*. *aeruginosa* biofilm matrices *in vivo* [39], it is assumed that exogenous gallium ions will be competing for EPS binding against pre-bound, “*native*”, calcium ions. As such, gallium accommodation by the mucoid EPS is modelled through calcium-for-gallium cation exchange into the 2-PolyMG/M systems. The initial 2-PolyMG/M structures are charge neutral and, therefore, calcium-for-gallium substitutions were charge-balanced to ensure the final complexed states were also charge neutral. In the 2-chain systems, two charged-balanced substitution mechanisms were investigated:

$$3\times Ca^{2+}(+6)\to 2\times Ga^{3+}(+6),$$ Scheme 1

$$4\times Ca^{2+}(+8)\to 2\times Ga^{3+}+2\times Na^{+}(+8).$$ Scheme 2

Note here, that **Scheme 1** retains a single native calcium ion within the EPS scaffold, whereas **Scheme 2** represents a full cation exchange. The thermodynamic stability of the exchange reaction was evaluated by means of calculating a formation energy according to **Eq 1**

$$E_{f}=E_{\left\{gallium-EPS\;complex\right\}}-(E_{\left\{EPS\right\}}+{l\mu }_{\left\{Na\right\}}+{m\mu }_{\left\{Ga\right\}}-{n\mu }_{\left\{Ca\right\}}).$$ (1)

Within **Eq 1**, *E*_{*gallium−EPS complex*}_ represents the final energy of the gallium-EPS complex, *E*_{*EPS*}_ represents the energy of the initial EPS system, *μ*_{*Na*}_ represents the chemical potential of sodium, *μ*_{*Ga*}_ represents the chemical potential of gallium and *μ*_{*Ca*}_ represents the chemical potential of calcium.

### EPS functionality most implicated in gallium binding

**Fig 1:**
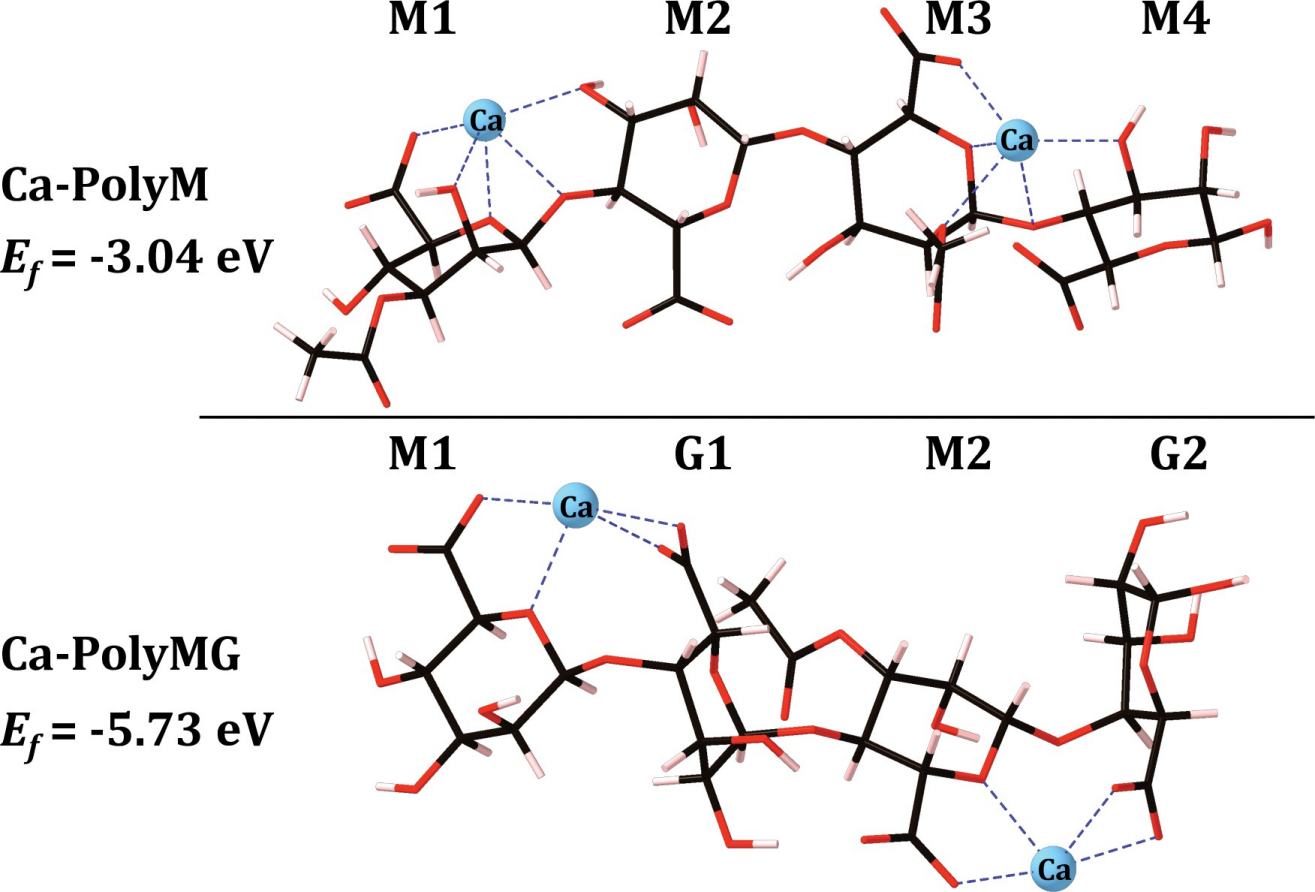
Mucoid EPS molecular models of acetylated poly-β-D-mannuronate fraction (1-PolyM; top) and acetylated copolymeric β-D-mannuronate-α-L-guluronate (1-PolyMG; bottom) at a 1-chain scale. Carbon atoms are shown in black, oxygen in red, hydrogen in pink. The native calcium ions are shown in blue and bonds to the calcium ions are also shown in blue. Uronate nomenclature is also given.

The calcium cross-linking sites in the 2-PolyMG and 2-PolyM systems are unique, each displaying different oxygen coordinating functionality, as well as coordination number (CN). Therefore, to better target the calcium-for-gallium substitutions in **Schemes 1 and 2**, initial DFT calculations were performed on a single EPS chain—a charge saturated calcium complexed 1-PolyMG/M system—to identify EPS functionality that best promoted gallium accommodation. The 1-PolyMG/M molecular models correspond to anionic (-4 charged) EPS quadramers, saturated by two Ca^2+^ ions and these systems are illustrated in **Fig 1**. Previous investigations within the group, identifying stable calcium ion complex geometries on a single mucoid (PolyMG/M) EPS chain, showed that the most stable calcium chelation geometries encompassed binding to hydroxyl, acetyl, ring, glycosidic and carboxylate oxygen donors and, additionally, it is 3–5 eV more thermodynamically stable to fully saturate the charge of the EPS backbone, compared to the binding of single ions within single M-M and M-G junctions [13]. As such, the calcium ion positions within the 1-PolyMG/M EPS systems (**Fig 1**) correspond to the most thermodynamically stable chelation sites for a charge saturating number of calcium ions along the length of a single PolyMG/M chain [13].

For the gallium substitutions into this 1-chain system, the following calcium-for-gallium charge-balanced substitution was investigated:

$$2\times {\mathrm{Ca}}^{2+}\to 1\times {\mathrm{Ga}}^{3+}+1\times {\mathrm{Na}}^{+}.$$ Scheme 3

### Water

Previous research has shown that cations interact with polysaccharides in positions that effectively exclude water molecules [50]. This is consistent with fluorescent lectin binding and confocal laser scanning microscopy studies that have shown that most of the water in the *in vivo P*. *aeruginosa* biofilm extracellular matrix, is confined to voids within the larger 3D architecture [51, 52]. Furthermore, NMR data have shown that water in the vicinity of polysaccharide chains is just as mobile as that further away, suggesting that very little water remains entrapped within the potential cation binding pockets [53] and any that is, would be rapidly exchanged with the hydrated environment. Consequently, we have not included water (either implicit or explicit) in these calculations, which also greatly reduced the computational burden of obtaining these data.

## Results and discussion

### 1-chain gallium stability

**Fig 2:**
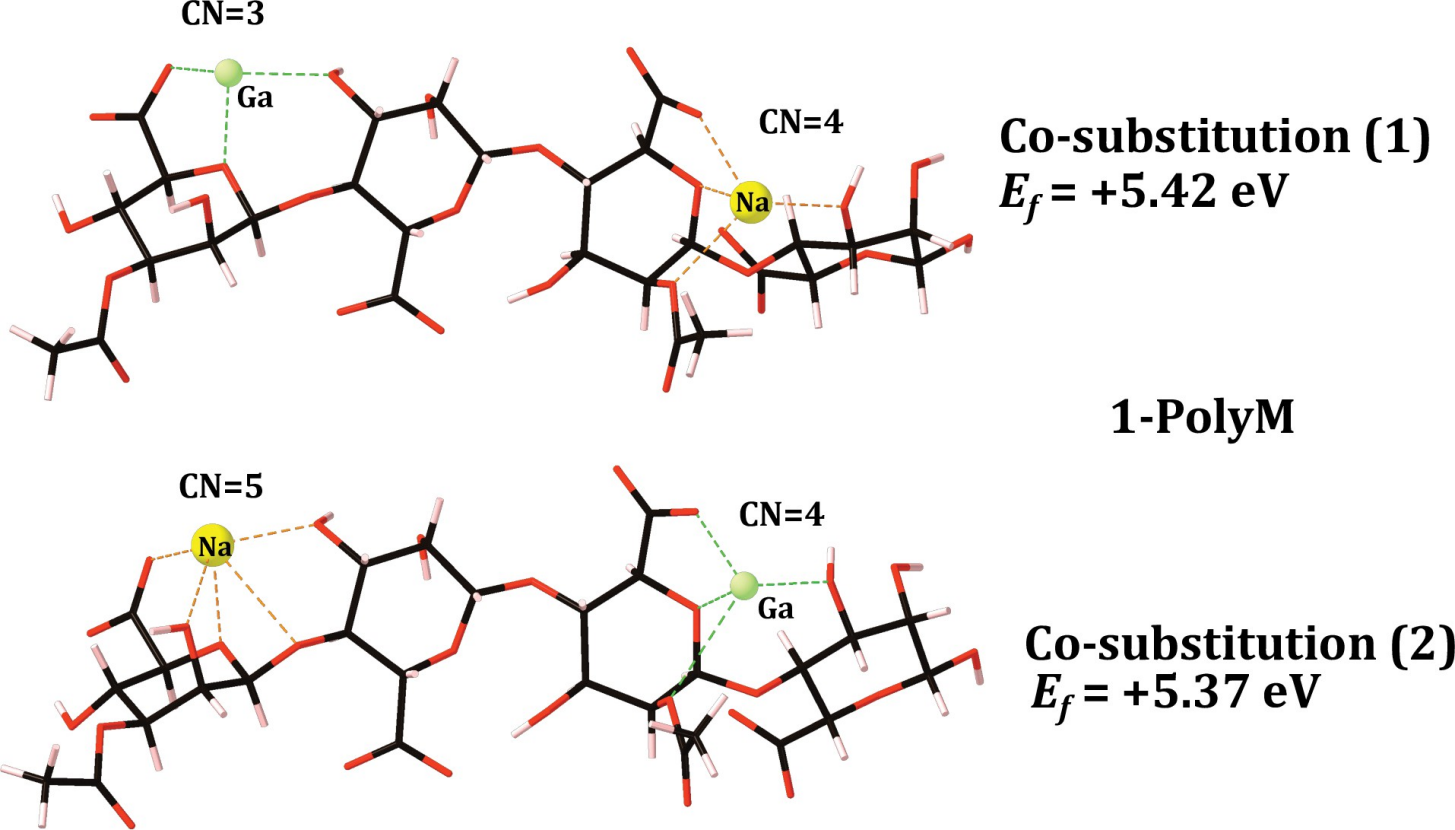
Optimised gallium 1-chain EPS complexes, along-side their formation energies (eV), for the 1-PolyM EPS scaffold. Carbon atoms are shown in black, oxygen in red, hydrogen in pink, gallium in green and sodium in yellow. Bonds to the gallium and sodium ions are shown as green and orange dashed lines respectively. The cation coordination numbers (CN) are also displayed. For reference the Ca Poly-M *Ef* is -3.04 eV (Fig 1).

**Fig 3:**
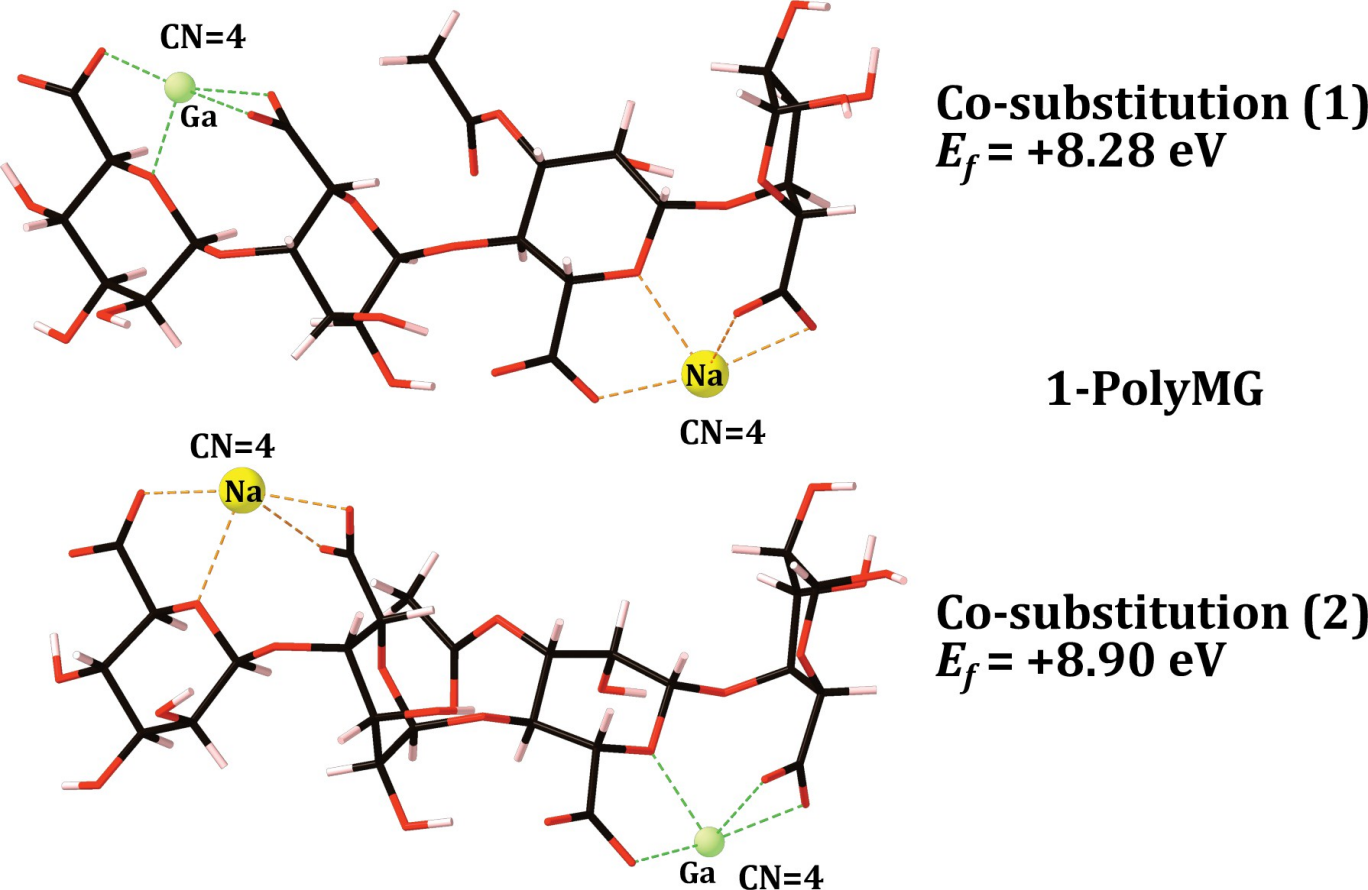
Optimised gallium 1-chain EPS complexes, along-side their formation energies (eV), for the 1-PolyMG EPS scaffold. Carbon atoms are shown in black, oxygen in red, hydrogen in pink, gallium in green and sodium in yellow. Bonds to the gallium and sodium ions are shown as green and orange dashed lines respectively. The cation coordination numbers (CN) are also displayed. For reference the Ca Poly-MG *Ef* is -5.73 eV (Fig 1).

The gallium exchange was performed at both unique calcium sites in both the 1-PolyM and 1-PolyMG scaffolds (**Figs 2** and **3**) and the formation energy for each co-substitution was calculated using **Eq 1**, where *E*_{*EPS*}_ represents the energy of the 1-PolyMG or 1-PolyM scaffold (**Fig 1**). The geometrical parameters for these complexes are given in **Table 1**.

**Table 1:** Average O-Ga bond lengths and Mulliken populations for the gallium 1-chain EPS complexes.

1-PolyM co-substitution (1)		
Bond	Average length (Å)	Average population (|e|)
COO^-^-Ga (monodentate)	2.01	0.19
OH-Ga	2.43	0.07
Ring O-Ga	2.20	0.13
1-PolyM co-substitution (2)		
COO^-^-Ga (monodentate)	1.97	0.24
OH-Ga	2.36	0.07
Ring O-Ga	2.19	0.14
Acetyl O-Ga	2.95	0.04
1-PolyMG co-substitution (1)		
COO^-^-Ga (monodentate)	1.84	0.36
COO^-^-Ga (bidentate)	1.98	0.19
Ring O-Ga	2.00	0.27
1-PolyMG co-substitution (2)		
COO^-^-Ga (monodentate)	1.90	0.25
COO^-^-Ga (bidentate)	2.14	0.11
Ring O-Ga	2.20	0.17

Firstly, it is important to note, that calcium-for-gallium substitutions into the 1-chain EPS scaffold are thermodynamically unstable (**Eq 1** returning a positive formation energy), indicating that gallium cannot be accommodated by a single EPS chain in preference to the native calcium ions. The origins of the instability are discussed in more detail in the following section, but this does not detract from the principle aim of these initial calculations—to identify EPS functionality that displays the *largest* gallium affinity. It is entirely plausible to observe a gain in thermodynamic stability as the number of coordinating EPS chains increases, as previously observed in the case of divalent cations [13].

The 1-chain gallium substitutions offer two critical geometrical insights. The first is that the gallium ion is prone to a drop in CN when complexed to the mannuronate rich scaffold, 1-PolyM (**Fig 2**). Relative to the native calcium, the CN drops by two for co-substitution (1), losing interactions with hydroxyl and glycosidic oxygens, and by one for co-substitution (2), losing interaction with the glycosidic oxygen (**Fig 2**). The ionic radius of Ga^3+^ (0.62 Å) is smaller than the ionic radius of Ca^2+^ (0.99 Å) [31, 54] and, consequently, it is not surprising to observe this drop in CN. However, intriguingly, gallium does not experience this same reduction to its CN when complexed into the 1-PolyMG scaffold (**Fig 3**). The primary structural feature distinguishing gallium (and calcium) complexation within single M-M and M-G junctions is the occurrence of two COO^-^ groups per cation in the latter compared to only one COO^-^ group in the former. The gallium ion can complex into a low energy geometry with a lower CN when more COO^-^ groups are involved in the complexation. This leads to the second geometrical insight, that the average COO^-^-Ga bond lengths are shorter, and Mulliken populations larger, than all other O-Ga contacts present within both the 1-PolyMG/M gallium complexes (**Table 1**). Explicitly, the COO^-^-Ga lengths are 0.16–1 Å shorter compared to all other O-Ga contacts and Mulliken populations indicate 1.5–6 time the charge transfer from COO^-^ donors relative to hydroxyl, ring and acetyl donors.

Taken collectively this work suggests the geometrical principle that COO^-^ groups are most implicated in gallium complexation on the EPS scaffold. This observation compliments recent DFT Gibbs free energy calculations, evaluating the spontaneity of substituting Ga^3+^ into Fe^3+^-metalloproteins, which also showed a strong stabilisation effect offered by carboxylate groups [55]. Specifically, the COO^-^ bearing amino acid residues, such as aspartate and glutamate, greatly increase Ga^3+^ selectivity compared to neutral amino acid residues such as histidine [55]. In addition, our finding is supported by thermogravimetric analyses that identified a gallium metal content of Ga^3+^-loaded alginate of 1.10 mmol/g, indicating a complete saturation of the COO^-^ groups alone [56]. This is not dissimilar to Fe^3+^ complexation by alginate fibres, where FTIR and X-ray photoelectronic spectroscopy (XPS) showed that COO^-^ is the only alginate functional group responsible for binding Fe^3+^ [57].

In light of this, the gallium substitutions into the 2-PolyMG/M systems (**Schemes 1 and 2**) were performed at the calcium sites that encompass the most COO^-^ donors and have the largest CNs.

### 2-chain calcium-for-gallium thermodynamic stability

**Fig 4:**
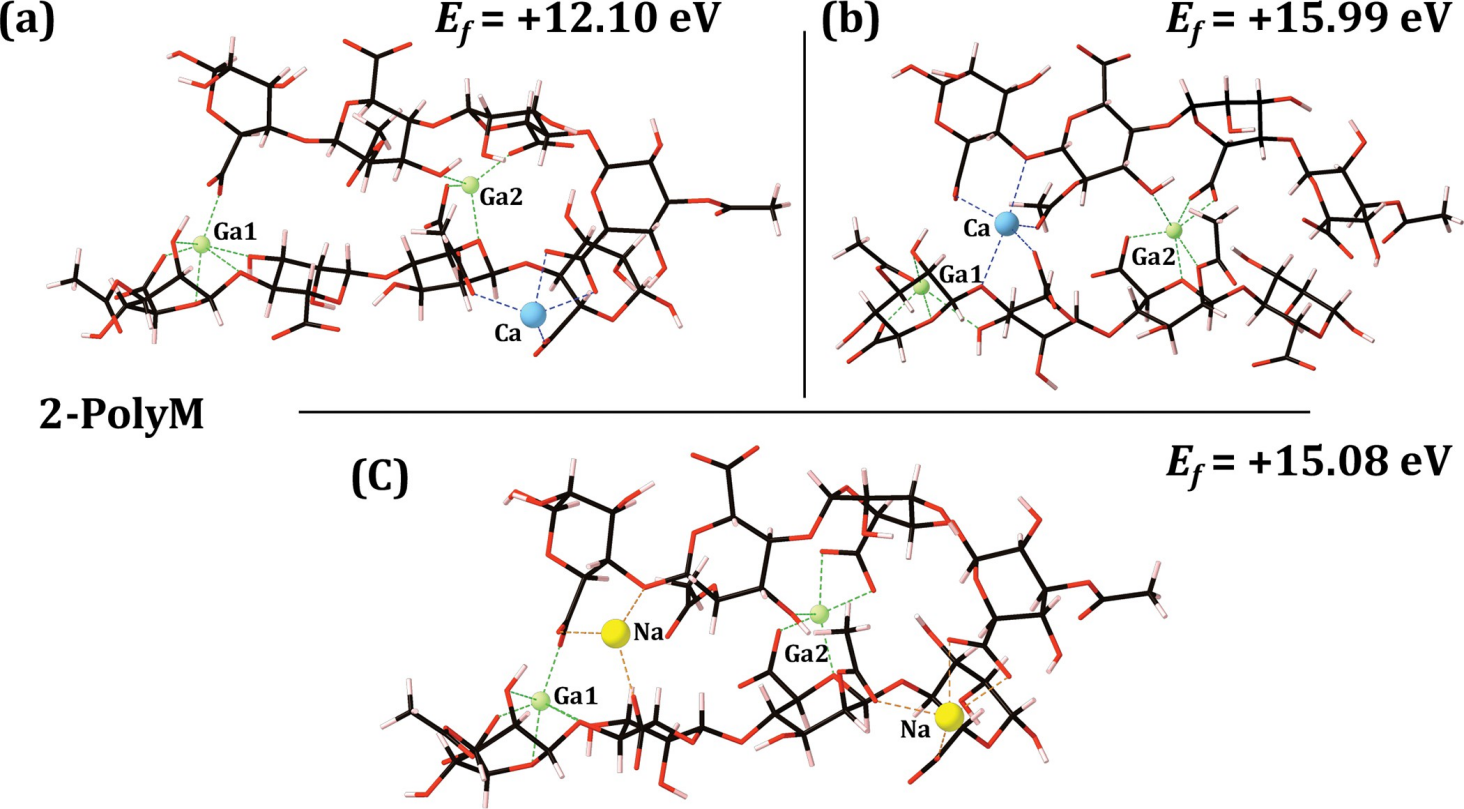
Optimised gallium 2-chain EPS complexes, along-side their formation energies (eV), for the 2-PolyM EPS scaffold. (a) substitution 1, (b) substitution 2, (c) substitution 3. Carbon atoms are shown in black, oxygen in red, hydrogen in pink, gallium in green and sodium in yellow. Bonds to the gallium and sodium ions are shown as green and orange dashed lines respectively. The gallium ions are labelled and the native calcium ions are shown in blue with bonds to these calcium ions also shown in blue. For reference the Ca-saturated 2-Poly-M *Ef* is -9.53 eV [13].

**Fig 5:**
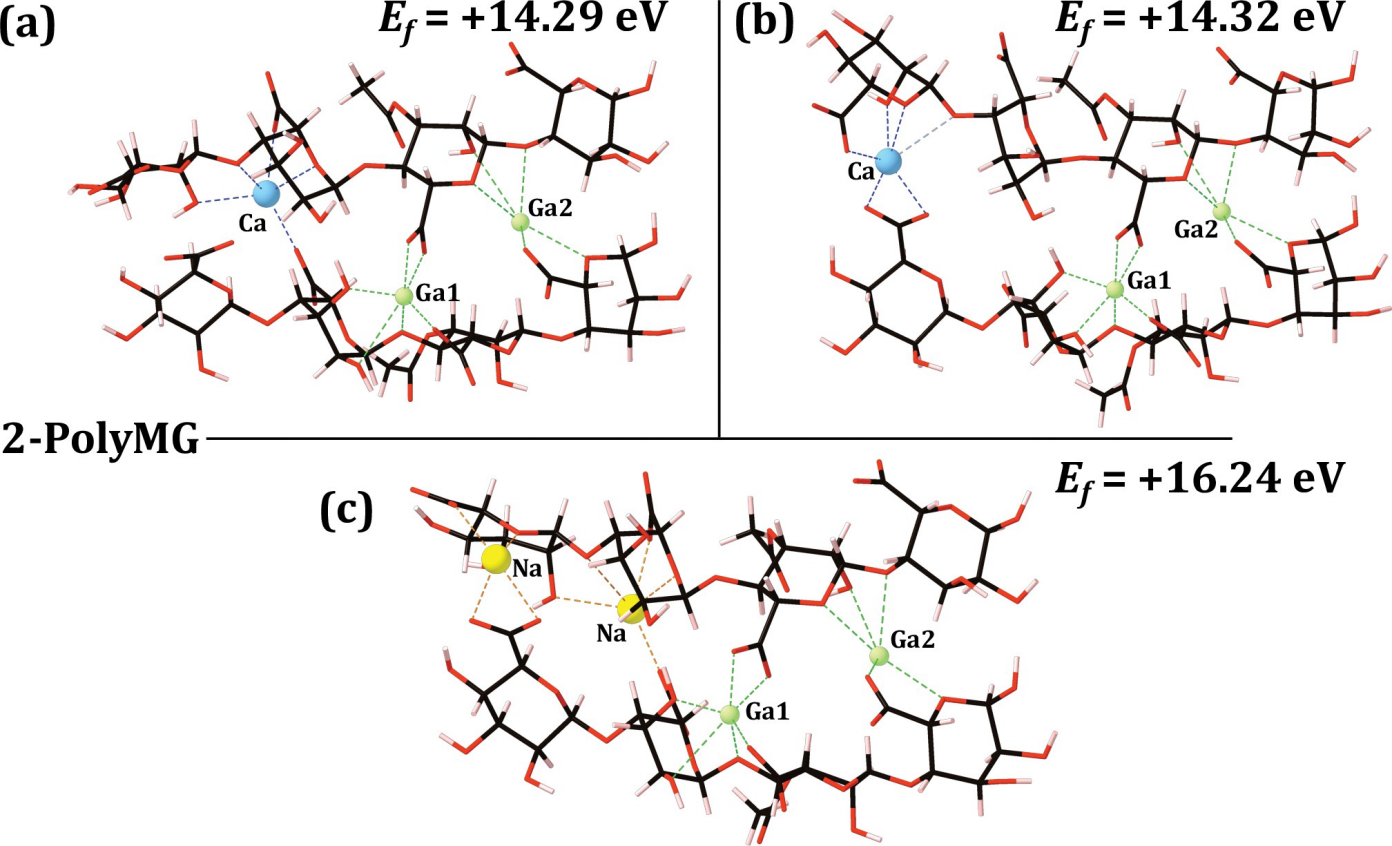
Optimised gallium 2-chain EPS complexes, along-side their formation energies (eV), for the 2-PolyMG EPS scaffold. (a) substitution 1, (b) substitution 2, (c) substitution 3. Carbon atoms are shown in black, oxygen in red, hydrogen in pink, gallium in green and sodium in yellow. Bonds to the gallium and sodium ions are shown as green and orange dashed lines respectively. The gallium ions are labelled and the native calcium ions are shown in blue with bonds to these calcium ions also shown in blue. For reference the Ca-saturated 2-Poly-M *Ef* is -10.01 eV [13].

The optimised gallium 2-chain EPS complexes, along-side their formation energies, for the 2-PolyM and 2-PolyMG EPS scaffolds, are given in **Figs 4** and **5** respectively.

The calcium-for-gallium cation exchange within the calcium cross-linked 2-chain EPS scaffolds is thermodynamically unstable in all cases. Increasing the number of substitutions in the coordinating EPS chains does not increase the stability of the substitutions, as it does for divalent cations [13]. Specifically, substitutions into the 2-chain EPS systems are more unfavourable by 7–10 eV for the PolyM scaffold and 6–8 eV for the PolyMG scaffold relative to their 1-chain analogues (**Figs 2** and **3**). Therefore, taken along-side the thermodynamic predictions of the 1-chain gallium complexes, it can be inferred that exogenous gallium will not displace the native calcium ions from their binding sites complexed within M-M and M-G (cross-linking) junctions.

Overall, there is a larger enthalpic barrier to the substitution in the 2-PolyMG system, which displays more positive formation energies than the 2-PolyM system. Substituting the gallium ions into the native calcium sites creates a charge separation, essentially co-localising two gallium ions on one half of the scaffold. To investigate whether this is the cause of the increased instability of the 2-PolyMG gallium complexes, relative to the 2-PolyM gallium complexes, an additional substitution scheme was assessed, whereby 1×Ga^3+^ + 1×Na^+^ are substituted at the high affinity sites leaving 2×Ca^2+^ retained within the scaffold. This now ensures that +4 charge is partitioned on each side of the 2-PolyMG scaffold. The optimised geometry for this complex is given in **S1 Fig**. This particular gallium substitution is thermodynamically unstable (*E*_*f*_ = + 7.57 eV); however, it is 5–8 eV and 7–9 eV more stable than the 2-PolyM and 2-PolyMG gallium complexes respectively (**Figs 4** and **5**). This suggests that an uneven distribution of positive charge across the EPS scaffold does contribute, to some degree, to the thermodynamic instability of the gallium substitution. Notwithstanding, it is not possible to attribute the instability solely to this phenomenon, as this particular complex (**S1 Fig**) remains thermodynamically unstable. This substitution retains two native Ca^2+^ ions within the EPS scaffold, rather than one (**Scheme 1**) or none (**Scheme 2**). Therefore, it must also be taken into consideration that, as fewer calcium ions are removed as part the exchange, the enthalpic barrier for calcium removal is reduced making the substitution, by virtue, more favourable. This is discussed in more detail below.

The gallium substitution patterns into the 2-chain EPS systems (**Figs 4** and **5**) are, effectively, accommodating a +3 ion in a -2 binding pocket. Therefore, to assess whether gallium can, in theory, be favourably complexed within a vacant -3 binding pocket, additional DFT calculations were performed, complexing Ga^3+^ into a tri-polyguluronate (non-acetylated algal alginate) quadramer scaffold. The gallium ions were bound exclusively at tri-carboxylate sites, as thermogravimetric analysis has identified that algal alginate complexes Ga^3+^ ions at COO^-^ sites alone [56]. The optimised gallium tri-polyguluronate complex is depicted in **S2 Fig** along-side the formation energy, evaluated according to **S1 File**. This system is weakly thermodynamically stable (*E*_*f*_ = -0.24 eV) indicating that Ga^3+^ ions can favourably complex into -3 binding pockets present within alginate trimers, aligning with previous DFT predictions [35]. Therefore, the -2 charge pocket generated upon liberation of the native calcium, appears to be insufficient to bind the incoming Ga^3+^ ion, despite charge neutrality being retained over the entire scaffold. In fact, the unsuitability of the 2-chain chelation sites for the gallium ion is further explored below.

### Gallium-EPS interactions

The geometrical parameters for the 2-PolyMG/M gallium complexes (**Figs 4** and 5) are given in **Tables 2** and 3. As with the 1-chain complexes, the COO^-^ group remains the EPS functional group offering the most stable mode of interaction with the alien gallium ion. Independent of the substitution position for both EPS scaffolds, the COO^-^-Ga interactions are most stable, experiencing average bond lengths up to 0.5 Å shorter, and average Mulliken populations up to 0.28 |e| larger, relative to the other O-Ga contacts across both 2-chain scaffolds (**Tables 2** and **3**). With the exception of the carboxylate donors, all O-Ga interactions are ionic in nature, possessing Mulliken populations < 0.25 |e|. The carboxylate donors, however, possess far larger Mulliken populations, reaching 0.4 |e| and 0.34 |e| in the 2-PolyM and 2-PolyMG gallium complexes respectively. This reveals a greater degree of charge transfer between the COO^-^ group and Ga^3+^ ion, giving rise to an interaction with a larger degree of covalency. Shorter COO^-^-M^3+^ bond lengths, compared to other uronate O-M^3+^ bond lengths, have also been observed in DFT modelling of trivalent metal cation (M^3+^ = Sc^3+^, La^3+^, Cr^3+^ Al^3+^, Fe^3+^ and Ga^3+^) interactions with non-acetylated algal alginate disaccharides, which also attribute the shorter COO^-^-M^3+^ lengths to larger degrees of covalency and, thus, more stable interactions [35].

**Table 2:** Average O-Ga bond lengths and Mulliken populations for the gallium 2-PolyM complexes.

2-PolyM substitution 1 (**Fig 4A**)		
Bond	Average length (Å)	Average population (|e|)
COO^-^-Ga (monodentate)	1.92	0.37
OH-Ga	2.01	0.25
Ring O-Ga	2.07	0.24
Glycosidic O-Ga	2.29	0.16
O-Ga (all O donors)	1.98	0.31
O-Ca (all O donors)	2.27	0.12
2-PolyM substitution 2 (**Fig 4B**)		
COO^-^-Ga (monodentate)	1.94	0.31
COO^-^-Ga (bidentate)	2.03	0.22
OH-Ga	2.57	0.10
Ring O-Ga	2.20	0.18
Acetyl O-Ga	2.37	0.19
O-Ga (all O donors)	2.24	0.19
O-Ca (all O donors)	2.37	0.13
2-PolyM co-substitution 3 (**Fig 4C**)		
COO^-^-Ga (monodentate)	1.87	0.40
COO^-^-Ga (bidentate)	1.99	0.23
OH-Ga	2.08	0.24
Ring O-Ga	2.10	0.22
Glycosidic O-Ga	2.17	0.18
O-Ga (all O donors)	2.02	0.27
O-Ca (all O donors)	-	-

**Table 3:** Average O-Ga bond lengths and Mulliken populations for the gallium 2-PolyMG complexes.

2-PolyMG substitution 1 (**Fig 5A**)		
Bond	Average length (Å)	Average population (|e|)
COO^-^-Ga (monodentate)	1.92	0.34
COO^-^-Ga (bidentate)	2.05	0.20
OH-Ga	2.58	0.13
Ring O-Ga	2.65	0.06
Glycosidic O-Ga	2.45	0.16
O-Ga (all O donors)	2.35	0.17
O-Ca (all O donors)	2.35	0.13
2-PolyMG substitution 2 (**Fig 5B**)		
COO^-^-Ga (monodentate)	1.92	0.34
COO^-^-Ga (bidentate)	2.05	0.19
OH-Ga	2.35	0.16
Ring O-Ga	2.60	0.06
Glycosidic O-Ga	2.39	0.17
O-Ga (all O donors)	2.27	0.18
O-Ca (all O donors)	2.41	0.09
2-PolyMG co-substitution 3 (**Fig 5C**)		
COO^-^-Ga (monodentate)	1.91	0.34
COO^-^-Ga (bidentate)	2.06	0.20
OH-Ga	2.50	0.14
Ring O-Ga	2.59	0.06
Glycosidic O-Ga	2.40	0.17
O-Ga (all O donors)	2.31	0.18
O-Ca (all O donors)	-	-

Also given in **Tables 2** and **3** are the average O-Ga and O-Ca lengths and populations, encompassing all oxygen donors, for the 2-PolyM and 2-PolyMG gallium complexes. For all complexes, the O-Ga populations are 0.04 |e| - 0.19 |e| larger relative to the O-Ca populations, indicating 1.31–2.5 times the charge transfer from the oxygen donors to the alien gallium ions relative to the native calcium ion [16]. On account of this, it is possible to distinguish uronate oxygen bonding to trivalent metal ions from uronate oxygen bonding to divalent metal ions by the larger degrees of covalency in O-ion contacts present within the former. The occurrence of O-Ga interactions with larger degrees of covalency is not specific to polysaccharide systems alone; gallium has also been observed to bind to regions of the gallium binding peptide C3.15 (NYLPHQSSSPSR) where donor lone pairs, for example in backbone carbonyl groups, establish larger magnitudes of charge transfer [58].

Although the alien gallium ions complex most strongly to COO^-^ groups, they do participate in binding to all types of oxygen donors, a property also observed in gallium-chitosan complexes and in gallium-alginate bio-glasses [59–61]. Contributions from the acetyl groups to the gallium chelate geometry is observed in the 2-PolyM gallium complexes (**Fig 4B** and **4C**), but not the 2-PolyMG gallium complexes. This is a distinguishing feature of the 2-PolyM system with regards to divalent cations [13] and is maintained here upon calcium-for-gallium exchange. In addition to acetyl contributions, 2-PolyM gallium complexation can be further differentiated from 2-PolyMG gallium complexation through analysis of the hydroxyl, glycosidic and ring O-Ga interactions. Within the 2-PolyM gallium complexes, the average OH-Ga and ring O-Ga contacts are 0.07–0.28 Å shorter relative to the glycosidic O-Ga interactions. Conversely, within the 2-PolyMG gallium complexes with the average glycosidic O-Ga contacts are now 0.1–0.21 Å *shorter* relative to the OH-Ga and ring O-Ga interactions (**Tables 2** and **3**).

For each cation chelate complex, there exists an average O-ion bond length as well as a number of satisfactory interactions (CN). As such, the volume of a given chelate pocket, which is not spherical, can be approximated by the average O-ion length divided by the CN. Although this estimate is a normalised length scale and not a strict volume measurement, it offers a good approximation to the change in volume upon the exchange, allowing an assessment of whether the chelate pocket is contracted or expanded upon calcium-for-gallium exchange. These normalised lengths are displayed in **S1 Table**. Although the O-Ga interactions occur at shorter length scales on average (**Tables 2** and **3**), it is interesting to observe that not all the gallium chelation geometries are contracted relative to the native calcium. In fact, contraction is only observed when two carboxylate groups are bound to the gallium ion. In this circumstance the chelation geometry is contracted by up to 0.12 Å across both 2-PolyMG/M gallium complexes. In contrast, when only a single carboxylate group is bound, the alien gallium chelation geometry is expanded relative to the native calcium chelate pocket, by up to 0.20 Å across both complexes, without observing an increase in CN. This further accentuates the role that COO^-^ groups play in tight gallium complexation to the EPS scaffold, specifically underscoring the need to establish ≥2 COO^-^ groups per gallium ion to maximise gallium affinity.

Analysis of the gallium-EPS interactions alone would suggest that substitution into the EPS scaffold, in principle, should be stable (viable) as the gallium ions complex with far greater covalent character. However, this is not the case and the origins of the thermodynamic instability are better identified by interrogating the torsional parameters, as well as the enthalpic barrier for calcium removal, in the following sections.

### Gallium induced EPS conformational change

When complexed in-between two EPS chains, the gallium ion is more susceptible, compared to 1-chain scaffolds (**Fig 3**), to a drop in CN, experiencing a reduction in CN across both 2-chain EPS scaffolds (**Figs 4** and **5**). The average reduction in the gallium CN, relative to the native calcium CN, is 1.33 and 1 for the 2-PolyM and 2-PolyMG gallium complexes respectively. The reduction in gallium CN is less for the PolyMG scaffolds as mannuronate-guluronate junctions offer more suitably oriented oxygen atoms to bind the smaller gallium ion. Torsion angles (*ϕ*, *ψ*)° across the mannuronate(M)-mannuronate(M) and mannuronate(M)-guluronate(G) junctions, present within the 2-PolyM and 2-PolyMG gallium complexes, are given in **S2** and **S3 Tables** respectively.

M-G junctions are more resistant to uronate backbone conformational change upon calcium-for-gallium cation exchange, with global changes in *ϕ* and *ψ* for gallium-complexed M-G junctions not exceeding 16° and 24° respectively. Whereas these changes exceed 22° and 57° respectively for gallium-complexed M-M junctions within the 2-PolyM gallium complexes. This also reinforces the observation that the M-G junctions offer suitably oriented oxygen functionality to bind the incoming gallium ion without the need for conformational rearrangement, explaining why the gallium CN for the 2-PolyMG complexes drops less severely compared to the 2-PolyM complexes. An additional large torsion change is observed across the top chain M3-M4 junction (Δ*ψ* = +45°), in the 2-PolyM gallium complex displayed in **Fig 4A** (substitution 1). This can be attributed to the vacant 2-chain cross-linking site exposed through the removal of a calcium ion. Consequently, this leaves the glycosidic oxygen vacant, which reorientates to face away from the molecular frame. This is visualized in **S3 Fig**. As such, the inference is that removal of calcium from the EPS scaffold, as opposed to binding gallium to the EPS scaffold, induces a larger conformational rearrangement in the uronate backbone. That gallium can only induce minor torsional change across the EPS backbone, principally occupying an unaltered native calcium chelate pocket, means it is unable to bring about conformational change required to mitigate against the drop in its CN.

**Fig 6:**
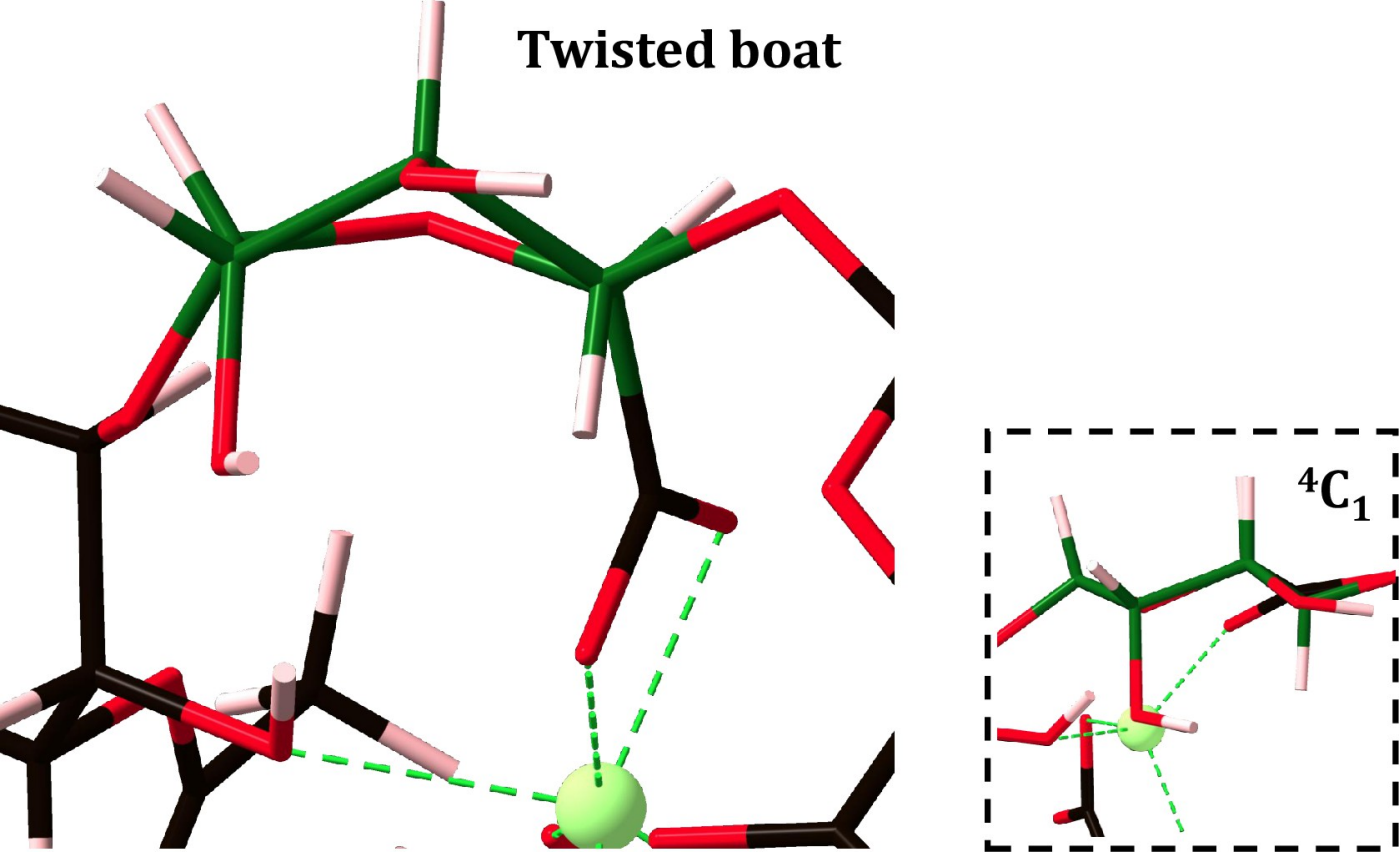
Less energetically favoured twisted-boat configuration adopted by the mannuronate residue (M2 top chain) within the 2-PolyM co-substitution gallium complex. Carbon atoms are shown in black, oxygen in red, hydrogen in pink and gallium in green. Bonds to gallium are shown in green. The twisted-boat uronate residue backbone is shown as dark green for clarity. For comparison, the ^4^C_1_ chair conformation is also given.

The 2-PolyM gallium complex hosts the first and third largest torsion changes, occurring across the top chain M2-M3 junction within the 2-PolyM substitution 2 (**Fig 4B**) and co-substitution 3 gallium complexes (**Fig 4C**), giving Δ*ψ* changes of +29° and +57° respectively. Compellingly, this occurs because the M2 uronate residue alters its conformation from the energetically stable chair (^4^C_1_) to the less energetically stable twisted-boat configuration as can be seen in **Fig 6**. This monosaccharide conformational change has also been observed in DFT structure predictions of trivalent metal ion complexes with non-acetylated algal alginate hexasaccharides [35]. At the single monosaccharide scale, this particular (pyranose) configuration lies approximately 5–8 Kcal/mol above the ground-state chair minimum [62]. Accordingly, it is therefore plausible that this particular conformational change serves to destabilize the gallium-EPS complex at a polysaccharide scale. In fact, by comparing the relative energy difference between a single PolyM EPS chain, in the absence of calcium ions, encompassing either the chair or twisted boat conformation at M2, it was calculated that the twisted boat conformation contributes a 4.58 eV instability to the quadramer conformation. It is, therefore, no surprise to observe that the 2-PolyM substitution 2 (**Fig 4B**) and co-substitution (**Fig 4C**) gallium complexes have reduced stability, approximately 3 eV more unstable relative to the 2-PolyM substitution 1 gallium complex (**Fig 4A**). 8 Kcal/mol (0.35 eV) falls below 4.58 eV and, as such, the mannuronate pseudorotation, namely the interconversion from chair to twisted boat conformation upon gallium exposure, causes greater destabilization at the polysaccharide (quadramer) scale relative to the monosaccharide scale. This particular EPS structural response is not observed in divalent ion-EPS complexation events [13].

### The primary calcium-for-gallium enthalpic barrier

Despite complexing with larger degrees of covalency, the gallium-EPS complexes remain highly thermodynamically unstable relative to the native (calcium-)EPS complexes. Interestingly, for both the 1-chain and 2-chain gallium-EPS complexes, it is more thermodynamically unstable to perform calcium-for-gallium cation exchanges across the PolyMG scaffolds, compared to the PolyM scaffolds. This observation holds true despite the larger drop in CN and destabilising gallium-induced mannuronate pseudorotation observed across the PolyM scaffolds.

Calcium ions display a 0.5 eV higher thermodynamic affinity for complexing within mannuronate-guluronate junctions on the EPS scaffold [13] and, expectedly, it is harder to liberate calcium ions from their chelate geometries within these junctions, relative to mannuronate-mannuronate junctions. In turn, this suggests that the stability of the initial calcium complex is the primary indicator of favourability for the calcium-for-gallium substitution rather than the stability of the resulting gallium complex. To further exemplify this point, calcium-for-gallium substitutions were introduced into a 4-chain PolyMG EPS system. Atomistic models of this system have previously been developed within the group and encompass six cross-linking calcium ions complexed in-between four parallel oriented, acetyl opposing, PolyMG scaffolds [40]. These cross-linking calcium ions are complexed within large CN environments (CN = 7) bound to three COO^-^ groups per ion, as well as to four uronate residues per ion [40]. This is in contrast to the three uronate residues per ion present within the 2-PolyMG EPS system. The formation energy, evaluated according to **Eq 1**, for the charge balanced exchange of all six cross-linking calcium ions for four gallium ions is +26.83 eV. This value indicates an extremely unfavourable substitution and gives rise to the following stability trend: no substitution > 1-chain EPS gallium complex > 2-chain EPS gallium complex > 4-chain EPS gallium complex. As the number of calcium cross-linked EPS chains increases, the calcium ions become increasingly encapsulated within the EPS network and, as such, harder to substitute for gallium. This, finally, gives rise to the observation that removal of calcium ions from their native (cross-linked) chelate pockets offers a large enthalpic barrier for the calcium-for-gallium cation exchange.

Taken collectively, the structural and thermochemical predictions of the 1-, 2- and 4-chain EPS gallium complexes indicate that exogenous gallium cannot be accommodated by the mature mucoid EPS through substitution of native calcium from its binding site on the mucoid EPS scaffold. This, therefore, suggests that intravenous gallium nitrate therapy (exogenous gallium) does not hinder mucoid *P*. *aeruginosa* CF biofilm proliferation through inducing calcium-for-gallium cation exchange or, therefore, creating an EPS morphology susceptible to physiological softening.

## Conclusion

Intravenous gallium therapy is emerging as a novel, non-antibiotic, therapeutic capable of inhibiting *P*. *aeruginosa* biofilm proliferation through siderophore quenching and represents a viable option to CF patients with chronic *P*. *aeruginosa* biofilm lung infections. The nature of any interactions between exogenous gallium ions and the native mucoid EPS scaffold has yet to be determined either experimentally or theoretically. Understanding such interactions will assist in elucidating an unknown mechanism of gallium therapy which assists in keeping gallium active against siderophore-deficient bacteria. To study gallium accommodation by the mature mucoid *P*. *aeruginosa* EPS, Density-Functional Theory (DFT) was deployed to ascertain whether gallium substitutions into the EPS scaffold in favour of the native calcium ions, which are responsible for facilitating and maintaining stable EPS architecture, were thermodynamically stable.

Upon substitution for the native calcium ions, the alien gallium ions receive larger degrees of charge transfer from EPS oxygen donors, forming interactions with larger degrees of covalency, relative to the native calcium ions. The COO^-^ group facilitates the largest degree of charge transfer to alien gallium ion, forming the most stable interactions, representing sites on the EPS scaffold with the largest gallium affinity. The increase in the covalent character of the oxygen-ion bonds is a key structural factor that allows divalent ion complexation by the EPS to be distinguished from trivalent ion complexation.

However, despite the gain in covalency upon substitution, it was shown that a charged balanced calcium-for-gallium cation exchange into the EPS scaffold was thermodynamically unfavourable at a 1-, 2- and 4-chain EPS scale. When performing calcium-for-gallium substitutions into the 2-chain EPS scaffolds, the gallium ion is susceptible to a drop in coordination number (CN) and cannot bring about conformational change large enough to mitigate against the drop in CN. Furthermore, in mannuronate rich EPS scaffolds, gallium can induce single mannuronate residues to adopt the less energetically stable twisted boat conformations. Both conformational features contribute to the instability of the substitution.

Finally, however, the largest enthalpic barrier to the calcium-for-gallium substitution is the removal of the native calcium ions from the EPS scaffold. Overall, the stability of the native calcium chelate geometry influences the favourability of the substitution more so than the stability of the resulting gallium complex. The thermodynamic predictions suggest that it is more stable for gallium to exist as an unbound ion in the EPS vicinity, which, encouragingly, highlights its retained availability for siderophore binding. Experimental validation of these results would be interesting, with ^1^H NMR being the prime candidate for success [63, 64]. Previous experiments exploring the calcium cross-linking of trisaccharides (from the marine sponge *Mircociona prolifera*), were effective, employing ^1^H NMR diffusion ordered spectroscopy (DOSY) to determine weak binding of calcium cations to the saccharide residues [64]. There is clearly potential for such an experiment to explore the Ca-Ga predications made here.

## Supporting information

S1 Fig Optimised gallium 2-chain EPS complex, along-side their formation energy (eV), for the 2-PolyMG EPS scaffold. This substitution pattern corresponds to 1×Ga^3+^ + 1×Na^+^ substituted at the high affinity sites leaving 2×Ca^2+^ retained within the scaffold, which ensures +4 charge is partitioned on each half of the 2-PolyMG scaffold. Carbon atoms are shown in black, oxygen in red, hydrogen in pink, gallium in green and sodium in yellow. Bonds to the gallium and sodium ions are shown as green and orange dashed lines respectively. The native calcium ions are shown in blue with bonds to these calcium ions also shown in blue.
(TIF)

S2 Fig Gallium accommodation by a tri-polyguluronate (non-acetylated algal alginate) quadramer scaffold with the associated formation energy. Carbon atoms are shown in black, oxygen in red, hydrogen in pink and gallium in green. Bonds to the gallium ions are shown as green dashed lines respectively.
(TIF)

S3 Fig Close up perspective of the native calcium chelate site (left) and the vacant coordination site created through substitution **Scheme 1** in the optimised gallium 2-PolyM complex displayed in **Fig 4A** (right). The *ψ* angle about the glycosidic linkage is displayed (green arrow) along-side the orientation of the glycosidic oxygen atom (black arrow). Carbon atoms are shown in black, oxygen in red, hydrogen in pink, gallium in green and calcium in.
(TIF)

S4 Fig PolyMG 4-chain system. (**a**) Gallium substitutions in the cross-linking calcium positions. (**b**) Calcium cross-linked 4-chain system. Only the cross-linking positions have been substituted by gallium and the two outer facing Ca-positions (marked in red on (b)) remain. The formation energies for both systems are marked. Carbon atoms are shown in black, oxygen in red, hydrogen in pink, gallium in green and calcium in blue. Bonds to the gallium and calcium ions are shown as green and blue dashed lines respectively. blue. Bonds to the gallium and calcium ions are shown as green and blue dashed lines respectively.
(ZIP)

S1 Table The normalised length scale within the native calcium and alien gallium. chelation pockets for 2-chain EPS scaffolds. The number of carboxylate groups bound to each gallium ion is reported also.
(DOCX)

S2 Table Torsion angles (*ϕ*, *ψ*)° across the mannuronate(M)-mannuronate(M) junctions in the 2-PolyM EPS systems and 2-PolyM gallium complexes. Uronate nomenclature is given in Fig 1. (DOCX)

S3 Table Torsion angles (*ϕ*, *ψ*)° across the mannuronate(M)-guluronate(G) junctions in the 2-PolyMG EPS systems and 2-PolyMG gallium complexes. Uronate nomenclature is given in **Fig 1**.
(DOCX)

S1 File In equation S1, *E*_{*Gallium*−*Polyguluronate*}_ represents the energy of the gallium tri-polyguluronate scaffold, *E*_{*Polyguluronic acid*}_ represents the energy of polyguluronic acid, *μ*_{*Ga*}_ is the chemical potential of gallium and *μ*_{*H*}_ is the chemical potential of a hydrogen atom. *μ*_{*H*}_ was calculated from the final energy of an optimised H_2_ molecule.
(DOCX)
