# Supplementary figures and images for: A DFT study of the gallium ion-binding capacity of mature Pseudomonas aeruginosa biofilm extracellular polysaccharide
Source: PLoS One. 2023 Jun 14;18(6):e0287191. doi: 10.1371/journal.pone.0287191 (PMC10266685; doi:10.1371/journal.pone.0287191)

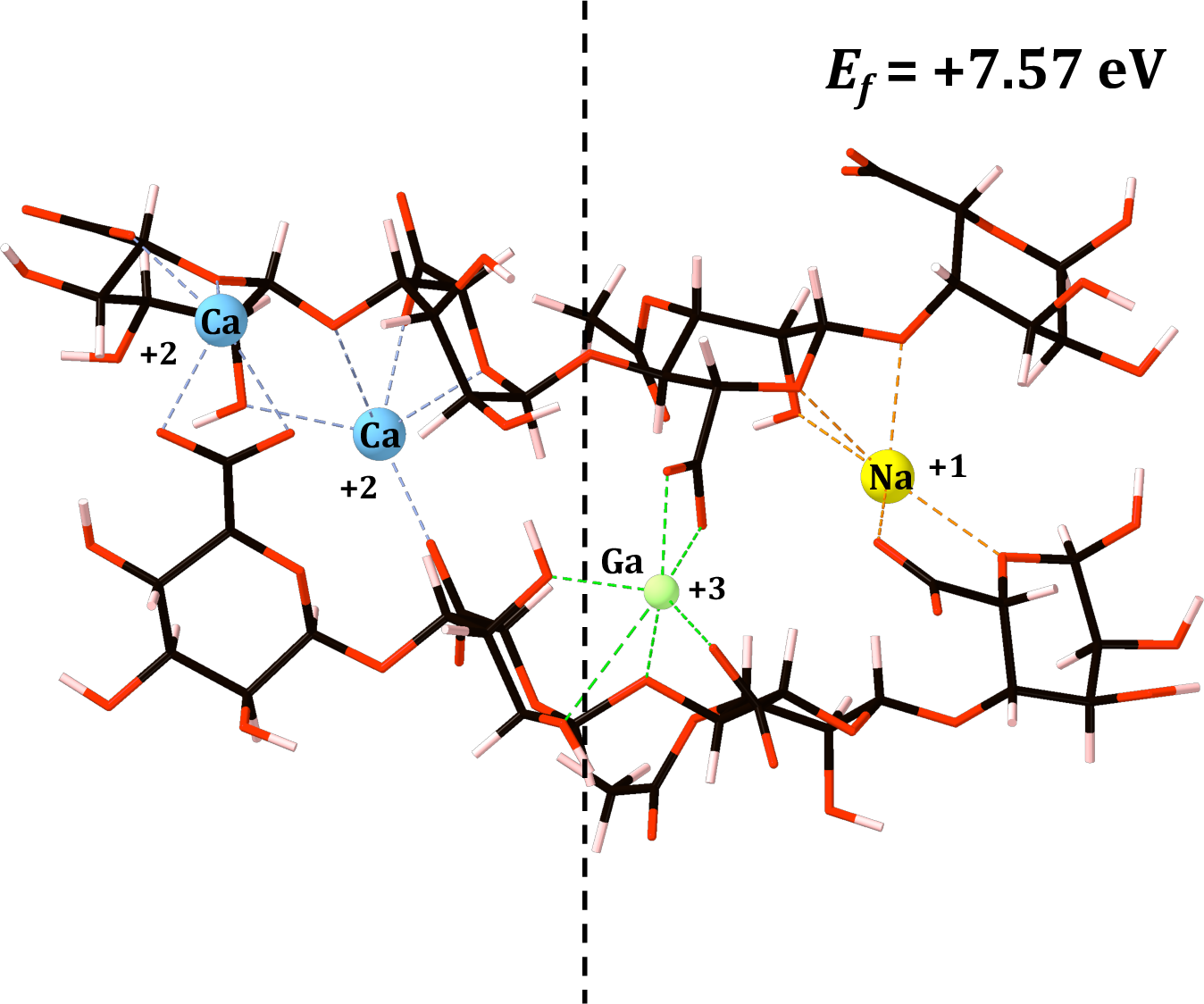

Supplement: S1 Fig — This substitution pattern corresponds to 1×Ga3+ + 1×Na+ substituted at the high affinity sites leaving 2×Ca2+ retained within the scaffold, which ensures +4 charge is partitioned on each half of the 2-PolyMG scaffold. Carbon atoms are shown in black, oxygen in red, hydrogen in pink, gallium in green and sodium in yellow. Bonds to the gallium and sodium ions are shown as green and orange dashed lines respectively. The native calcium ions are shown in blue with bonds to these calcium ions also shown in blue. (TIF) [file pone.0287191.s001.tif]

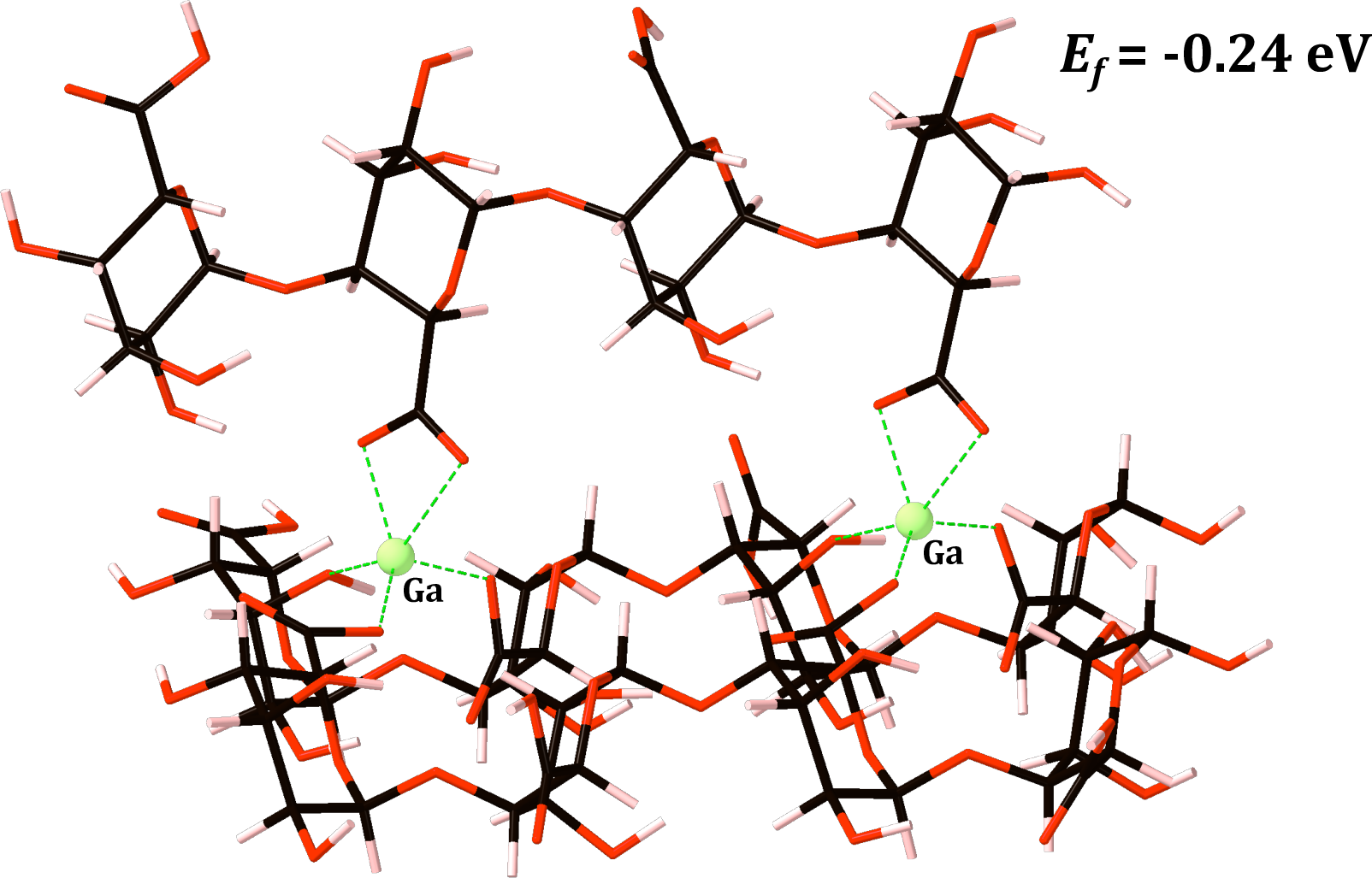

Supplement: S2 Fig — Carbon atoms are shown in black, oxygen in red, hydrogen in pink and gallium in green. Bonds to the gallium ions are shown as green dashed lines respectively. (TIF) [file pone.0287191.s002.tif]

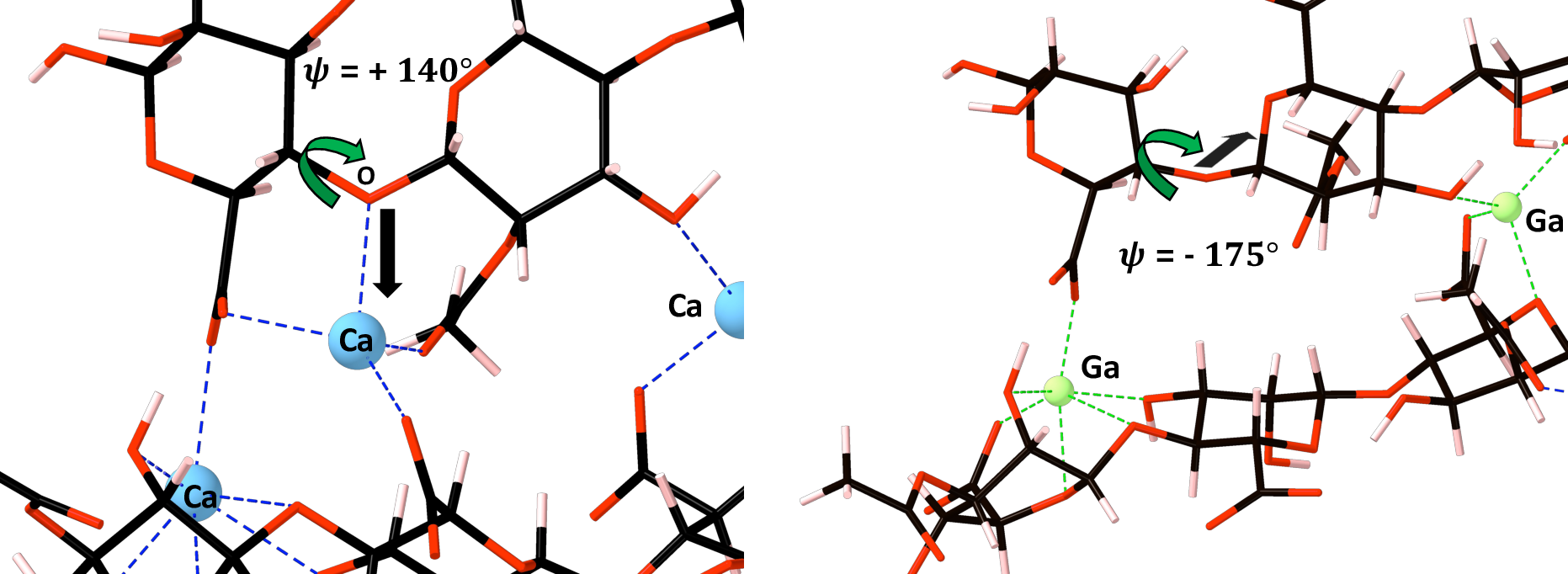

Supplement: S3 Fig — Close up perspective of the native calcium chelate site (left) and the vacant coordination site created through substitution Scheme 1 in the optimised gallium 2-PolyM complex displayed in Fig 4A (right). The ψ angle about the glycosidic linkage is displayed (green arrow) along-side the orientation of the glycosidic oxygen atom (black arrow). Carbon atoms are shown in black, oxygen in red, hydrogen in pink, gallium in green and calcium in. (TIF) [file pone.0287191.s003.tif]

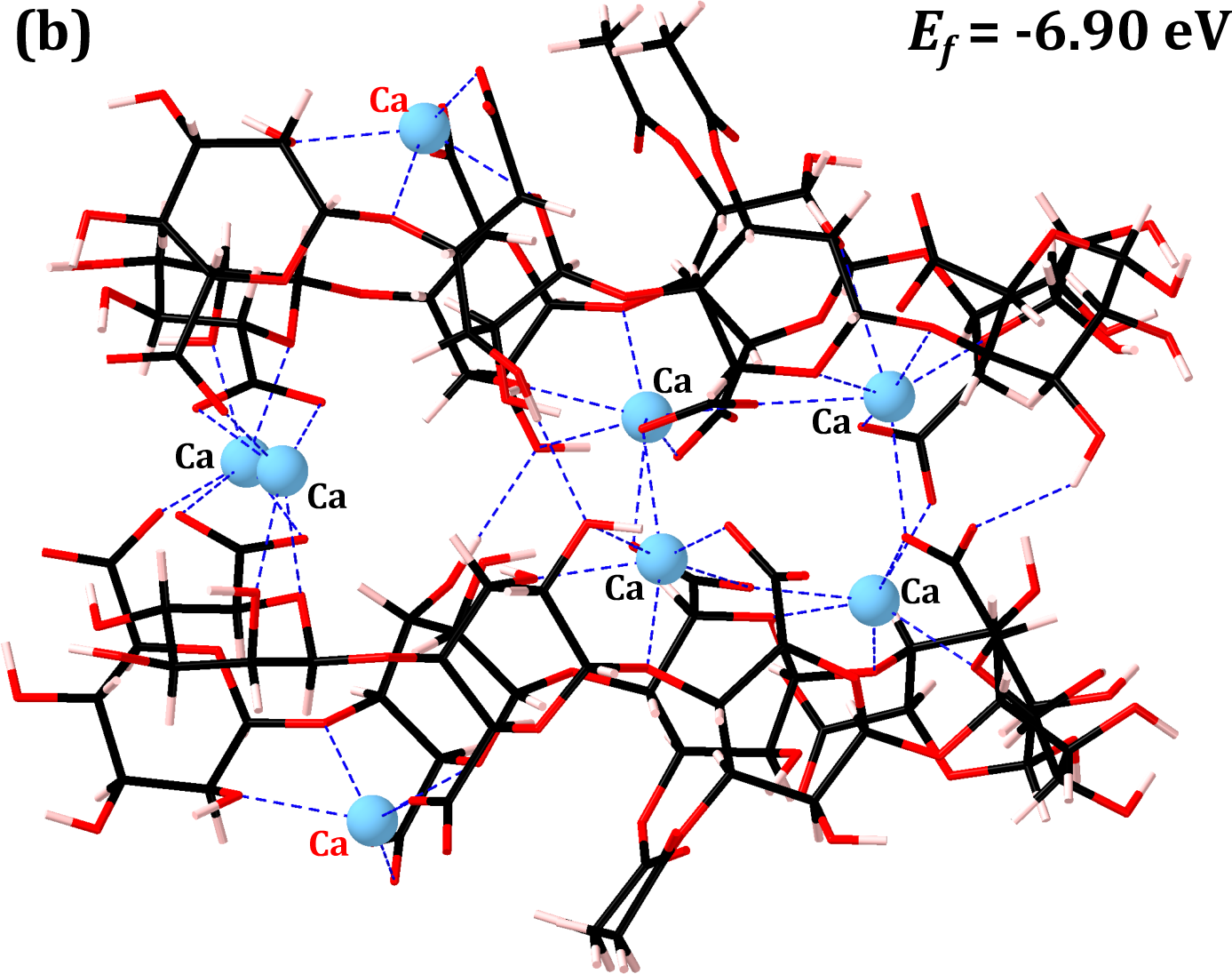

Supplement: S4 Fig — (a) Gallium substitutions in the cross-linking calcium positions. (b) Calcium cross-linked 4-chain system. Only the cross-linking positions have been substituted by gallium and the two outer facing Ca-positions (marked in red on (b)) remain. The formation energies for both systems are marked. Carbon atoms are shown in black, oxygen in red, hydrogen in pink, gallium in green and calcium in blue. Bonds to the gallium and calcium ions are shown as green and blue dashed lines respectively. blue. Bonds to the gallium and calcium ions are shown as green and blue dashed lines respectively. (ZIP) [file pone.0287191.s004.zip › FigS4b.tif]

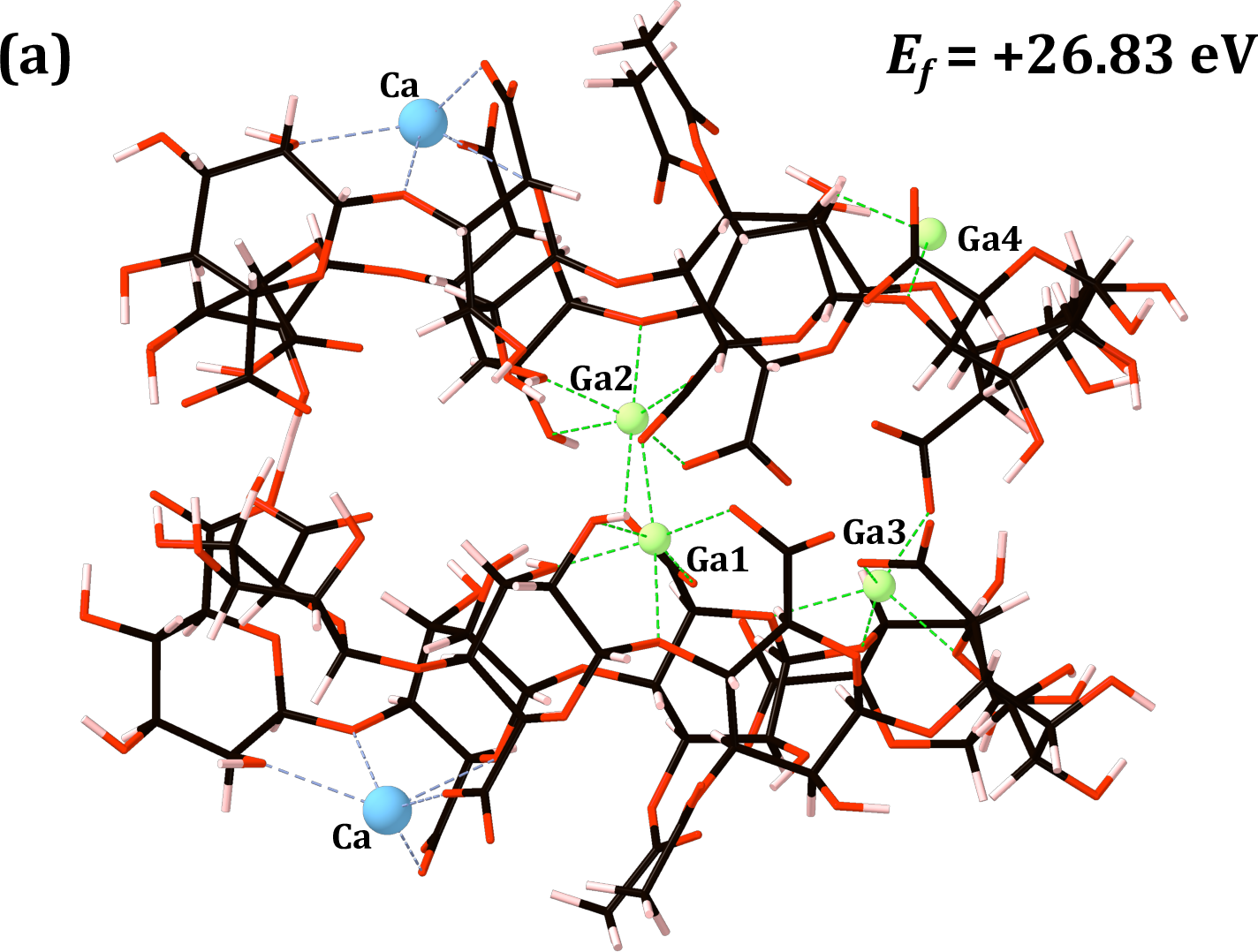

Supplement: S4 Fig — (a) Gallium substitutions in the cross-linking calcium positions. (b) Calcium cross-linked 4-chain system. Only the cross-linking positions have been substituted by gallium and the two outer facing Ca-positions (marked in red on (b)) remain. The formation energies for both systems are marked. Carbon atoms are shown in black, oxygen in red, hydrogen in pink, gallium in green and calcium in blue. Bonds to the gallium and calcium ions are shown as green and blue dashed lines respectively. blue. Bonds to the gallium and calcium ions are shown as green and blue dashed lines respectively. (ZIP) [file pone.0287191.s004.zip › FigS4a.tif]
