# Supplementary material for: A DFT study of the gallium ion-binding capacity of mature Pseudomonas aeruginosa biofilm extracellular polysaccharide
Source: PLoS One. 2023 Jun 14;18(6):e0287191. doi: 10.1371/journal.pone.0287191 (PMC10266685; doi:10.1371/journal.pone.0287191)
Supplement: S1 Table — chelation pockets for 2-chain EPS scaffolds. The number of carboxylate groups bound to each gallium ion is reported also. (DOCX) [file pone.0287191.s005.docx]

**Supporting information for:**

**A DFT study of the gallium ion-binding capacity of mature *Pseudomonas aeruginosa* biofilm extracellular polysaccharide**

Oliver J. Hills^1*^, Zuzanna Poskrobko^1^, Andrew J. Scott^2^, James Smith^1^ & Helen F. Chappell^1*^

^1^School of Food Science & Nutrition, University of Leeds, Woodhouse Lane, Leeds, LS2 9JT, UK

^2^School of Chemical & Process Engineering, University of Leeds, Woodhouse Lane, Leeds, LS2 9JT, UK

* Corresponding author

Email: [H.F.Chappell@leeds.ac.uk](mailto:H.F.Chappell@leeds.ac.uk) (HFC)

**Average lengths (Å) of the native calcium ions in their respective chelation pockets bound to the 2-PolyM and 2-PolyMG EPS scaffolds prior to their removal as part of the cation exchange.**

**Table S1**: The normalised length scale within the native calcium and alien gallium

chelation pockets for 2-chain EPS scaffolds. The number of carboxylate groups bound to each gallium ion is reported also.

| Normalised length scale within the chelate pocket (Å) | | | | |
| --- | --- | --- | --- | --- |
|  | O-Ca1 [1] | O-Ga1 | O-Ca2 [1] | O-Ga2 |
| 2PolyM substitution 1 | 0.41 | 0.34  2 COO^-^/Ga | 0.47 | 0.47  2 COO^-^/Ga |
| 2PolyM substitution 2 |  | 0.61  1 COO^-^/Ga |  | 0.35  2 COO^-^/Ga |
| 2PolyM co-substitution |  | 0.34  2 COO^-^/Ga |  | 0.40  2 COO^-^/Ga |
| 2PolyMG substitution 1 | 0.40 | 0.35  2 COO^-^/Ga | 0.41 | 0.52  1 COO^-^/Ga |
| 2PolyMG substitution 2 |  | 0.34  2 COO^-^/Ga |  | 0.43  1 COO^-^/Ga |
| 2PolyMG co-substitution |  | 0.35  2 COO^-^/Ga |  | 0.43  1 COO^-^/Ga |

**References**

1. Hills OJ, Smith J, Scott AJ, Devine DA, Chappell HF. Cation complexation by mucoid Pseudomonas aeruginosa extracellular polysaccharide. Deshpande PA, editor. PLoS One [Internet]. 2021 Sep 2 [cited 2021 Sep 6];16(9):e0257026. Available from: https://journals.plos.org/plosone/article?id=10.1371/journal.pone.0257026
