# Supplementary material for: A DFT study of the gallium ion-binding capacity of mature Pseudomonas aeruginosa biofilm extracellular polysaccharide
Source: PLoS One. 2023 Jun 14;18(6):e0287191. doi: 10.1371/journal.pone.0287191 (PMC10266685; doi:10.1371/journal.pone.0287191)
Supplement: S2 Table — (DOCX) [file pone.0287191.s006.docx]

**Supporting information for:**

**A DFT study of the gallium ion-binding capacity of mature *Pseudomonas aeruginosa* biofilm extracellular polysaccharide**

Oliver J. Hills^1*^, Zuzanna Poskrobko^1^, Andrew J. Scott^2^, James Smith^1^ & Helen F. Chappell^1*^

^1^School of Food Science & Nutrition, University of Leeds, Woodhouse Lane, Leeds, LS2 9JT, UK

^2^School of Chemical & Process Engineering, University of Leeds, Woodhouse Lane, Leeds, LS2 9JT, UK

* Corresponding author

Email: [H.F.Chappell@leeds.ac.uk](mailto:H.F.Chappell@leeds.ac.uk) (HFC)

**Torsional parameters for the gallium 2-chain EPS complexes.**

**Table S2**: Torsion angles ($\phi, \psi$)$^{\circ}$ across the mannuronate(M)-mannuronate(M) junctions in the 2-PolyM EPS systems and 2-PolyM gallium complexes. Uronate nomenclature is given in **Fig 1**.

| System | M1-M2 ($\phi, \psi$)$^{\circ}$ | M2-M3 ($\phi, \psi$)$^{\circ}$ | M3-M4 ($\phi, \psi$)$^{\circ}$ |
| --- | --- | --- | --- |
| 2-PolyM top chain | (-51, -99) | (-51, -127) | (-49, +140) |
| 2-PolyM bottom chain | (-76, -121) | (-94, -104) | (-102, -109) |
| 2-PolyM substitution 1 top chain | (-53, -105) | (-63, -123) | (-73, -175) |
| 2-PolyM substitution 1 bottom chain | (-72, -107) | (-85, -154) | (-94, -112) |
| 2-PolyM substitution 2 top chain | (-53, -93) | (-59, -98) | (-57, +144) |
| 2-PolyM substitution 2 bottom chain | (-76, -127) | (-84, -102) | (-106, -108) |
| 2-PolyM co-substitution 3 top chain | (-48, -119) | (-73, -70) | (-23, +136) |
| 2-PolyM co-substitution 3 top chain | (-80, -120) | (-91, -119) | (-99, -98) |
