# Supplementary material for: A DFT study of the gallium ion-binding capacity of mature Pseudomonas aeruginosa biofilm extracellular polysaccharide
Source: PLoS One. 2023 Jun 14;18(6):e0287191. doi: 10.1371/journal.pone.0287191 (PMC10266685; doi:10.1371/journal.pone.0287191)
Supplement: S3 Table — Uronate nomenclature is given in Fig 1. (DOCX) [file pone.0287191.s007.docx]

**Supporting information for:**

**A DFT study of the gallium ion-binding capacity of mature *Pseudomonas aeruginosa* biofilm extracellular polysaccharide**

Oliver J. Hills^1*^, Zuzanna Poskrobko^1^, Andrew J. Scott^2^, James Smith^1^ & Helen F. Chappell^1*^

^1^School of Food Science & Nutrition, University of Leeds, Woodhouse Lane, Leeds, LS2 9JT, UK

^2^School of Chemical & Process Engineering, University of Leeds, Woodhouse Lane, Leeds, LS2 9JT, UK

* Corresponding author

Email: [H.F.Chappell@leeds.ac.uk](mailto:H.F.Chappell@leeds.ac.uk) (HFC)

**Table S3**: Torsion angles ($\phi, \psi$)$^{\circ}$ across the mannuronate(M)-guluronate(G) junctions in the 2-PolyMG EPS systems and 2-PolyMG gallium complexes. Uronate nomenclature is given in **Fig 1**.

| System | M1-G1 ($\phi, \psi$)$^{\circ}$ | G1-M2 ($\phi, \psi$)$^{\circ}$ | M2-G2 ($\phi, \psi$)$^{\circ}$ |
| --- | --- | --- | --- |
| 2-PolyMG top chain | (-91, -127) | (-94, -130) | (-110, -78) |
| 2-PolyMG bottom chain | (-58, -122) | (-89, -130) | (-64, -44) |
| 2-PolyMG substitution 1 top chain | (-84, -129) | (-86, -120) | (-101, -86) |
| 2-PolyMG substitution 1 bottom chain | (-55, -130) | (-98, -147) | (-62, -49) |
| 2-PolyMG substitution 2 top chain | (-105, -103) | (-90, -144) | (-102, -78) |
| 2-PolyMG substitution 2 bottom chain | (-58, -117) | (-89, -144) | (-56, -47) |
| 2-PolyMG co-substitution 3 top chain | (-97, -104) | (-86, -128) | (-99, -86) |
| 2-PolyMG co-substitution 3 top chain | (-56, -120) | (-97, -148) | (-62, -50) |
