# Supplementary material for: A DFT study of the gallium ion-binding capacity of mature Pseudomonas aeruginosa biofilm extracellular polysaccharide
Source: PLoS One. 2023 Jun 14;18(6):e0287191. doi: 10.1371/journal.pone.0287191 (PMC10266685; doi:10.1371/journal.pone.0287191)
Supplement: S1 File — μ{H} was calculated from the final energy of an optimised H2 molecule. (DOCX) [file pone.0287191.s008.docx]

**Supporting information for:**

**A DFT study of the gallium ion-binding capacity of mature *Pseudomonas aeruginosa* biofilm extracellular polysaccharide**

Oliver J. Hills^1*^, Zuzanna Poskrobko^1^, Andrew J. Scott^2^, James Smith^1^ & Helen F. Chappell^1*^

^1^School of Food Science & Nutrition, University of Leeds, Woodhouse Lane, Leeds, LS2 9JT, UK

^2^School of Chemical & Process Engineering, University of Leeds, Woodhouse Lane, Leeds, LS2 9JT, UK

* Corresponding author

Email: [H.F.Chappell@leeds.ac.uk](mailto:H.F.Chappell@leeds.ac.uk) (HFC)

**Gallium substitution into tri-polyguluronate (non-acetylated algal alginate) quadramer scaffold.**

$$E_{f}=E_{\left\{ Gallium-Polyguluronate \right\}}-\left( {3E}_{\left\{ Polyguluronic acid \right\}}+2\mu_{\left\{ Ga \right\}}-6\mu_{\left\{ H \right\}} \right) (S1)$$

**Eq S1:** In equation S1, $E_{\left\{ Gallium-Polyguluronate \right\}}$ represents the energy of the gallium tri-polyguluronate scaffold, $E_{\left\{ Polyguluronic acid \right\}}$ represents the energy of polyguluronic acid, $\mu_{\left\{ Ga \right\}}$ is the chemical potential of gallium and $\mu_{\left\{ H \right\}}$ is the chemical potential of a hydrogen atom. $\mu_{\left\{ H \right\}}$ was calculated from the final energy of an optimised H_2_ molecule.
